# 

**Published:** 1860-07

**Authors:** 


### CONTENTS OF HALL'S JOURNAL OF HEALTH FOR JULY.

(New-York, $1 a year.)

Athletics, .......... 145
Central Park, .......... 147
Size of Parks, .......... 149
The Teeth, .......... 150
Physical Training, .......... 152
Water Filters, .......... 159
Dyspeptic Letters, .......... 160
Two Best Doctors, .......... 162
Notices, Reviews, etc., .......... 163
Book Notices, .......... 164

